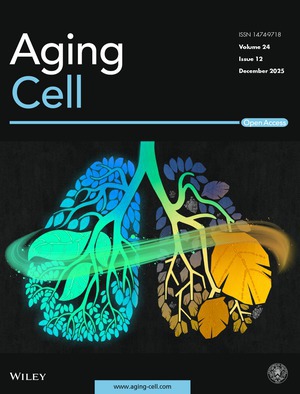# Featured Cover

**DOI:** 10.1111/acel.70324

**Published:** 2025-12-08

**Authors:** Xinying Zeng, Jingya Li, Jiaxin Wang, Jiaxin Zhang, Yuhua Wang, Yan Wang, Yifei Wang, Lin Tian, Zhonghui Zhu

## Abstract

Cover legend: The cover image is based on the article *Matrix Stiffness Promotes DRP1‐Mediated Myofibroblast Senescence to Drive Silica‐Induced Pulmonary Fibrosis* by Xinying Zeng et al., https://doi.org/10.1111/acel.70275.